# The role of self-regulating abilities in long-term weight loss in severely obese children and adolescents undergoing intensive combined lifestyle interventions (HELIOS); rationale, design and methods

**DOI:** 10.1186/1471-2431-13-41

**Published:** 2013-03-25

**Authors:** Jutka Halberstadt, Sabine Makkes, Emely de Vet, Anita Jansen, Chantal Nederkoorn, Olga H van der Baan-Slootweg, Jacob C Seidell

**Affiliations:** 1 Department of Health Sciences and the EMGO Institute for Health and Care Research, VU University Amsterdam, Amsterdam, the Netherlands; 2 Faculty of Psychology and Neuroscience, Department of Clinical Psychological Science, Maastricht University, Maastricht, the Netherlands; 3 Merem Treatment Centers, Heideheuvel, Hilversum, the Netherlands

**Keywords:** Pediatric, Obesity, Weight loss, Lifestyle, Psychology, Determinants

## Abstract

**Background:**

Adequate treatment of severe childhood obesity is important given its serious social, psychological and physical consequences. Self-regulation may be a crucial determinant of treatment success. Yet, little is known about the role that self-regulation and other psychosocial factors play in the long-term outcome of obesity treatment in severely obese children and adolescents.

In this paper, we describe the design of a study that aims to determine whether the ability to self-regulate predicts long-term weight loss in severely obese children and adolescents. An additional objective is to identify other psychosocial factors that may modify this relation.

**Methods/design:**

The study is designed as a prospective observational study of 120 severely obese children and adolescents (8–19 years) and their parents/caregivers undergoing an intensive combined lifestyle intervention during one year. The intervention uses behavior change techniques to improve the general ability to self-regulate.

Measurements will be taken at three points in time: at baseline (start of treatment), at the end of treatment (1 year after baseline) and at follow-up (2 years after baseline). The primary outcome measurement is the gender and age-specific change in SDS-BMI.

The children’s general self-regulation abilities are evaluated by two behavioral computer tasks assessing two distinct aspects of self-regulation that are particularly relevant to controlling food intake: inhibitory control (Stop Signal Task) and sensitivity to reward (Balloon Analogue Risk Task). In addition to the computer tasks, a self-report measure of eating-specific self-regulation ability is used. Psychosocial factors related to competence, motivation, relatedness and outcome expectations are examined as moderating factors using several questionnaires for the patients and their parents/caregivers.

**Discussion:**

This study will provide knowledge about the relation between self-regulation and long-term weight loss after intensive lifestyle interventions over a two-year period in severely obese children and adolescents, a growing but often overlooked patient group. We aim to investigate to what extent (changes in) the general ability to self-regulate predicts weight loss and weight loss maintenance. This study will also contribute to the knowledge on how this association is modified by other psychosocial factors. The results may contribute to the development of more successful interventions.

**Trial registration:**

Netherlands Trial Register (NTR1678, registered 20-Feb-2009)

## Background

### Prevalence and consequences of obesity

The prevalence of obesity in the Netherlands increased 6-fold in the period 1980–2009 in boys (0.3% to 1.8%) and 4.5-fold in girls (0.5% to 2.2%) [1]. Generally, when there is an increase in prevalence of obesity, there is a greater relative increase in severe obesity [2].

Childhood and adolescent obesity are associated with serious comorbidities including type 2 diabetes mellitus, hyperlipidemia, hypertension, respiratory and musculoskeletal conditions and liver abnormalities [3-5]. The increase in obesity-associated diseases leads to a significant increase in direct and indirect medical costs [6]. In addition to physical health problems, obese children and adolescents also are more likely to suffer from a variety of psychosocial problems [7-9]. They are more likely than non-obese children to be a target of societal stigmatization, including teasing and bullying [10,11], to be socially isolated [5,12], to have relatively high rates of disordered eating, anxiety, and depression [5], and to suffer from suicidal thoughts and making suicide attempts [9]. When they reach adulthood, they are less likely than their thinner counterparts to complete college and more likely to live in poverty [5]. They are also less likely to get married [13]. A further illustration that obesity has a large impact on young people’s lives is reflected in the finding that severely obese children and adolescents reported to have similar quality of life as those diagnosed with cancer [14,15]. Therefore adequate management of severe childhood obesity may contribute to reduce their current and future social, psychological and physical impairment.

### Intensive combined lifestyle interventions

It is generally recognized that the more severe forms of obesity may well warrant more intensive therapeutic interventions [16] than less severe obesity [17]. Because regular outpatient treatment appears to be insufficiently effective for the specific patient group of severely obese children and adolescents [18-20], it has been proposed that there is a need for experienced, specialized pediatric obesity centers that can provide intensive treatment by a multidisciplinary team with expertise in childhood obesity and its comorbidities [18-20]. According to several guidelines, the treatment team should include a physician, dietician, exercise specialist and psychologist or other mental health care provider that is able to offer behavioral counseling [17-22].

A promising alternative to regular outpatient treatment is so called “immersion treatment” that places patients in a therapeutic and educational environment for extended periods of time, for example a residential summer camp or inpatient setting [21,23]. Immersion programs described in a recent review included the components controlled diet, physical exercise/activity, nutrition education and therapy and/or education regarding behavior change. The participants in the reviewed treatments that included a follow up lost an average of 23.9% of their overweight during treatment and 20.6% pre-immersion to follow up (ranging from 4 months to 3.6 years later) [23]. Inclusion of cognitive behavioral therapy (CBT), defined as including “regular group and/or individual meetings with a therapist utilizing CBT techniques for managing behavior change, such as self-monitoring, motivational interviewing/decisional counseling and problem-solving”, seems especially promising, resulting in an average of 29.9% loss of overweight in total at follow-up, compared to 9.4% for programs without cognitive behavioral therapy [23].

Heideheuvel (part of Merem Treatment Centers) is a specialized clinic in the Netherlands offering a form of immersion treatment, by means of an intensive inpatient combined lifestyle intervention, focusing on nutrition, physical activity and behavior change of the severely obese participants and their parents. Improving self-regulation of eating behavior is one of the main goals of the treatment at Heideheuvel. The clinic uses cognitive behavioral techniques to improve self-regulation.

Although the need for combined lifestyle interventions targeting nutrition, physical activity and behavior change is widely acknowledged, long-term follow-up studies of obesity interventions are lacking, especially for severely obese youth [17,24,25].

According to Yanovski and Yanovski the known long-term results for children and adolescents are generally disappointing, because the weight reduction is often not maintained [18]. However, there appear to be remarkable individual differences in treatment success [26]. For some patients treatment is highly successful, while others continue to gain weight despite treatment. This raises the question what determines inter-individual variability in intervention success.

Currently there is little insight in the psychosocial factors that may be crucial in determining the long-term outcome. The ability to self-regulate dietary intake has been proposed as an important factor in weight loss and weight loss maintenance [8,27-32].

### The role of self-regulation

Severe obesity results from a sustained chronic positive energy balance. This implies that there is an underlying inability to regulate food intake in such a way that it matches energy expenditure. Volkow and others have postulated that this inability to regulate food intake can be seen as a brain-related dysfunction whereby reward-driven urges for food override the cognitive ability to limit food intake [33]. Especially children and adolescents are vulnerable to problems arising due to self-regulatory failure, because the neurocognitive structures that link reward systems to the executive control system are still in development [34]. The inability to self-regulate is particularly problematic for children who are overwhelmed with an abundance of food and food cues due to their socio-economic and cultural environments or who grow up in families where the parents have insufficient parenting skills to teach their children self-regulation in response to food cues [35].

Self-regulation encompasses any, conscious and non-conscious, efforts by people to alter their thoughts, emotions, attention, impulses and behavior [36] in the service of attaining and maintaining personal goals [37]. Self-regulation reflects the ability to resist immediate rewards (e.g. a chocolate cake) in the face of long-term goal pursuit (e.g. losing weight and maintaining the weight loss) [38]. It is known that people differ greatly in their ability to self regulate [39].

Not many studies examined self-regulation of food intake in obese individuals, but the few studies that did consistently showed that obese people generally are less able to self-regulate than lean people [27,28,40,41].

Two distinct aspects of self-regulation that are particularly relevant to controlling food intake, are sensitivity to reward and inhibitory control [40,42,43]. Sensitivity to reward is associated with the mesolimbic dopamine system [44]. It reflects the sensory pleasure associated with the reward and the motivation to obtain the reward. Inhibitory control is regulated by the prefrontal cortex, and refers to the executive function by which impulses or responses are controlled [43,44].

Research has indeed indicated that food is more rewarding for overweight children than for lean children, making it therefore harder for them to resist food temptations and possibly increasing the chance of excessive food intake and resulting further weight gain [45,46]. Obese children are found to have a higher sensitivity to reward and less response inhibition than lean children [29,41,47-52]. For example, Nederkoorn and colleagues showed that obese children had lower levels of inhibitory control as assessed by a behavioral measure for disinhibition and were more sensitive to reward as assessed by a response preservation measure than leaner children [40]. Above these cross-sectional studies, prospective studies showed that differences in ability to self-regulate were related to weight gain. For example Francis and colleagues showed that children who were less able to self-regulate at ages 3 and 5 years, as measured with behavioral laboratory tasks, had a more rapid weight gain from age 3 to 12 years [51].

Poor self-regulation, as measured with various questionnaires and behavioral measures, has also been shown to predict less weight loss, less weight loss maintenance or more attrition to weight loss programs [27,28,30,40,53]. For example, those obese children participating in a cognitive behavioral treatment program who showed relatively little inhibitory control lost less weight than those who exhibited higher levels of control [28].

Three decades ago Bonato et al. already suggested that interventions for obese children should aim at improving self-regulation of eating [29]. More recent research indeed indicates that self-regulation of eating can be improved through behavioral treatment. For example, Israel et al. evaluated an intervention for overweight children and their parents that aimed to improve self-regulation in children in order to lose weight. Training components included instructions for goal setting, formulating and implementing a plan to change behavior, self-evaluation, self-reward and training in problem-solving behaviors appropriate for high-risk or tempting situations. The results indicated that improving self-regulation can help to maintain long term weight loss [31]. Bryant et al. also indicated in a review that disinhibition in eating behavior can be successfully diminished through application of behavioral therapy aimed at self-regulation of eating behavior [27].

In sum, relatively poor self-regulation is likely to contribute to the development of obesity as well as to a lower amount of long-term weight loss as a result of treatment. Therefore, studying the role of self-regulation in the effectiveness of weight loss therapy may contribute to the development of more successful interventions [28]. The main objective of this study is to determine whether the ability to self-regulate predicts long-term weight loss in severely obese children and adolescents. To our knowledge, such studies have not been performed in severely obese children and adolescents.

### The potential moderating role of other psychosocial factors

An additional objective of this study is to identify other psychosocial factors that may modify the relation between the general ability to self-regulate and long-term weight loss in severely obese children and adolescents. Gaining understanding in moderating factors is important as it might help to improve tailoring interventions to children. The following factors that plausibly play an important role will be assessed:

1) Competence, in this study operationalized as general self-efficacy and self-worth.

2) Motivation, in this study operationalized as autonomous motivation.

3) Relatedness, in this study operationalized as interaction between parent and child, peer body size, social competences and social problems, parental feeding style and affect of the parent.

4) Outcome expectations, in this study operationalized as the difference between current and expected own body size.

The selection of these additional psychosocial factors to study, was made based on 1) reviews by Teixeira et al. [26] and Elfhag and Rössner [54], 2) advice by experts in the field of psychological child obesity research and treatment and 3) two prevailing psychological theories: self-determination theory [55] and social cognitive theory [56].

The self-determination theory and the social cognitive theory are general theories of human behavior, but have also been applied to weight control. According to self-determination theory, a theory of human motivation and behavior, three innate psychological needs are the basis for autonomous motivation: 1) competence (i.e. having a feeling of efficacy), 2) autonomy (i.e. perceiving an internal locus of causality; having a feeling of free will) and 3) relatedness (i.e. having a sense of security and belonging) [55]. Conditions in the social context, like positive or negative feedback, can either enhance or hinder the fulfillment of these three basic needs [55]. When satisfied, these psychological needs facilitate autonomous motivation, which is important for behavioral persistence in for example weight-related behaviors [55,57].

According to social cognitive theory human behavior is a result of a continuous reciprocal interaction between behavior, cognitive and affective personal factors and environmental events [56,58]. This interaction is influenced by people’s beliefs about their capabilities to exercise control over their own level of functioning and over events that affect their lives [56,59]. These self-efficacy beliefs determine people’s level of motivation and the effort they are willing to put in reaching their goals [56,59]. Self-efficacy influences outcome expectations which has an effect on the motivation to perform: when you expect to succeed that is an incentive to pursue the needed actions [56].

Some of the factors from the self-determination theory and the social cognitive theory are also mentioned in reviews by Teixeira et al. and Elfhag and Rössner [26,54] on psychosocial factors that are associated with weight control in adults. These reviews for example show that more autonomous motivation is associated with weight loss maintenance [26,54]. Other factors that are mentioned are: self-efficacy [26,54], self-esteem [26], autonomy [26,54], social support [54], body image [26] and outcome expectations [26,54].

In sum, the objective of this study is mainly to determine whether the ability to self-regulate predicts long-term weight loss in severely obese children and adolescents and in addition to identify other psychosocial factors that may modify the relation between the general ability to self-regulate and long-term weight loss.

It is hypothesized that having less self-regulating abilities will result in less weight loss and less weight loss maintenance. The following factors are expected to negatively influence the relationship between self-regulation ability and weight loss and weight loss maintenance: less general self-efficacy, lower self-worth, less autonomous motivation, lower quality of the relationship between parent and child, larger body size of peers, less social competences, more social problems, a less adequate parental feeding style, more negative affect of the parent and unrealistic outcome expectations.

## Methods/design

### Study design

This study is designed as a prospective observational study of children and adolescents undergoing an intensive combined lifestyle intervention during one year for their severe obesity. The treatment program has either a 2 months or a 6 months inpatient period.

The Medical Ethics Committee of VU University Medical Center Amsterdam has approved the study design, protocols and informed consent procedure. This study is a collaboration with a study that aims to provide insights into the effectiveness and cost-effectiveness of the interventions [60].

### Setting

This study is carried out in the childhood obesity clinic Heideheuvel (part of Merem Treatment Centers) in Hilversum, The Netherlands between August 2009 and July 2013.

### Study population

The aim is to include 120 children and adolescents and their parents/caregivers in the study. 80 of the patients will undergo a 2 months inpatient period and 40 a 6 months inpatient period.

Patients (8–19 years of age) are admitted to Heideheuvel for their severe obesity. Inclusion criteria are a SDS-BMI ≥ 3.0 according to the growth curves based on the fourth Dutch National Growth Study of 1997 (this corresponds to the 99.9th percentile) or a SDS-BMI ≥ 2.3 (corresponding to the 99th percentile) with obesity related morbidity (e.g. obstructive sleep apnea syndrome, hyperinsulinemia, diabetes type 2, liver function disorders, dyslipidemia, musculoskeletal problems). Before admission, these children and adolescents have, unsuccessfully, received outpatient obesity care elsewhere. Some patients, who are diagnosed to not be able to profit from outpatient care, are referred directly. All patients are referred by their local pediatrician.

Criteria for exclusion from the study are: syndromal/chromosomal determined obesity, obesity caused by endocrine disorders (e.g. hypothyroidism, Cushing syndrome, primary hyperinsulinemia, pseudohypoparathyroidism, acquired (structural) hypothalamic damage) or use of medication (e.g. antiepileptic drugs, antidepressants), psychiatric disorders (e.g. severe depression, schizophrenia) that may obstruct adequate treatment, presence of eating disorders (binge eating disorder, bulimia nervosa) to such a degree that specific therapeutic attention is needed before starting the intervention, children/adolescents or parents that can or will not give ‘informed consent’, parents that can or will not participate in the treatment, children/adolescents with an IQ below 75 or attending a school for intellectually challenged children. Written informed consents are obtained from both the participants (from age 12) and their parents.

Participants do not pay for the treatment which is temporarily reimbursed by the Ministry of Health, Welfare and Sports. For their participation in the research they get reimbursed with a 20 Euros gift voucher to cover travel expenses.

### Time schedule

The study period is four years. Since the capacity to treat patients is limited in the clinic, the patients are treated in sequential groups of 10 children (8–13 years) or 10 adolescents (13–19 years). At the end of the fourth year 120 patients will have completed the program and follow up period.

Measurements will be taken at three points in time: at baseline (start of treatment; T0), at the end of treatment (12 months after baseline; T12), at the end of follow-up (24 months after baseline; T24). Besides weight and height, measured at T0, T12 and T24, measurements include (also for the parents of the patients) several questionnaires on psychosocial characteristics: Dutch Eating Behavior Questionnaire for children (administered at T0, T12, T24), General self-efficacy scale (T0, T12, T24), Self-Perception Profile for Children/Adolescents (T0, T12, T24), Treatment Self-Regulation Questionnaire (T0), parent and child version of the Parent–child Interaction Questionnaire (T0), Child Behavior Checklist (T0, T12, T24), Parental Feeding Style Questionnaire (T0, T12, T24), Positive and Negative Affect Schedule (T0), line drawings of silhouettes to choose from (T0, T12, T24). In addition the children’s self-regulation abilities are evaluated by two behavioral computer tasks: The Stop Signal Task (T0, T12, T24) and the Balloon Analogue Risk Task (T0, T12, T24). All questionnaires and computer tasks are described in the measurements section below.

### Interventions

The treatment is an intensive one year lifestyle intervention program by a multidisciplinary team with emphasis on nutrition, exercise and behavior and implementation of the learned behavior in the home situation. Treatment consists of group treatment as well as individual sessions. For an extensive description of the treatment components, see Makkes et al. [60].

There is active and frequent participation of the parents during the whole treatment because parents have a major influence on their children’s weight and weight-related behaviors through modeling and encouraging of appropriate health behaviors, controlling the quality and quantity of the food available in the household, facilitating physical activity, and engaging in appropriate feeding practices. An additional advantage of family-based interventions is that they can improve health behaviors and weight not only in the treated child but also in siblings and adults living in the household [61,62].

The behavioral part of the treatment program is carried out by psychologists, social workers and group coaches in individual as well as group sessions with children and parents together and separately. The program uses behavior change techniques, e.g. from cognitive behavioral therapy, to improve the general ability to self-regulate. This improvement in general self-regulation abilities is assumed to facilitate the self-regulation of the newly learned healthier behavior. For example, the program teaches patients to delay gratification (I want a cookie now, but I will wait until it is tea time), to plan behavior (e.g. forming if-then plans: if it rains, I will still take my planned thirty minute walk) or to self monitor behavior (keeping a diary of the cookies eaten and the walks taken). The treatment further addresses: disordered eating behaviors, self-worth, self-efficacy, behavioral and emotional problems, autonomous motivation, body image, outcome expectations, mood disorders, eating and exercise behavior, interaction between parent and child, parental feeding styles, relationships with peers, body acceptance and coping. To achieve its aims, the program applies a number of behavior change techniques. These techniques can be specified with a 40-item taxonomy, called CALO-RE, that is developed by Michie et al. [63]. The chief psychologist of the treatment program scored the intervention with this CALO-RE taxonomy, which gives an overview of relevant behavior change techniques for interventions aimed at increasing physical activity and healthy eating [63]. The treatment program uses 27 of the 40 defined behavior change techniques to some extent (number 1–13, 15–16, 19, 21–22, 28–29, 33, 35–40). For example, the program uses goal setting to define a desired behavior that can be related to changes in diet or physical activity. Goal setting is also used to define health outcomes. Another used behavior change technique is prompting generalization of a target behavior from the clinic to the home situation. To learn how to cope with barriers to change the behavior, like certain feelings and desires, the treatment uses barrier identification and problem solving techniques. The treatment also uses modeling of behavior, which involves showing how to perform a certain behavior in for example a role play, by the staff, the peers and the parents. Another applied technique is motivational interviewing in which the staff is trained and that is used to assess and if necessary help change the phase of behavior change the patient is in.

### Measurements

#### Outcome measures

The primary outcome measurement is the gender and age-specific ΔSDS-BMI between baseline (T0) and follow up (T24) indicating total weight loss from baseline to follow up and ΔSDS-BMI between end of treatment (T12) and follow up (T24) indicating weight loss maintenance after treatment.

#### Determinants of long-term weight loss and weight loss maintenance

##### Behavioral measures of general self-regulation ability

Our hypothesis is that a primary determinant of (sustained) weight loss is the general ability to self-regulate. We assess two separate aspects of general self-regulation: sensitivity to reward and inhibitory control. Both are measured with two computerized behavioral tasks that are not food related and an objective measure of the general ability to self-regulate: the Stop Signal Task and the Balloon Analogue Risk Task [64,65].

The Stop Signal Task is a measure of inhibitory control [66] also referred to as executive response inhibition [67]. The task correlates significantly with related measures for self-control [66]. A Dutch adaption of the task by C. Nederkoorn was used [68]. The Stop Signal Task is a computer task consisting of a series of 4x64 trials, preceded by two practice blocks of 8 and 16 trials, in which individuals are exposed to a colored quadrangle on the right or the left side of the screen (the go signal). Individuals have to press the shift button on the side where the quadrangle appears as quick as possible. However, in 25% of the trials respondents hear a tone, after which individuals should inhibit their response to press the shift button. Failing to inhibit the response when the stop signal sounds, indicates lack of self-regulation. At the start of the task the delay between the go signal and the stop signal is set to 250 milliseconds. The computer changes the stop signal delay after every stop signal trial. Following an unsuccessful inhibition the delay is decreased by 50 milliseconds, making the task easier. After a successful inhibition the task is made more difficult by increasing the stop signal delay by 50 milliseconds. This dynamic adjustment results in a stop signal delay that enables the participant to stop on approximately 50% of the stop trials. The main outcome is the stop signal reaction time (SSRT), which is calculated by subtracting the stop signal delay from the mean go signal reaction time, measured in milliseconds. A longer SSRT means that the participant takes longer to inhibit his/her response and indicates less inhibitory control [64]. The Stop Signal Task demonstrated less inhibitory control in children with impulse control problems like ADHD [64,69] and in obese children [28,70], making it a valid test to study self-regulation in the present sample as well.

The Balloon Analogue Risk Task (BART) is a measure of sensitivity to reward [65], which has been shown to correlate significantly with related measures of self-control [66] and impulsivity [65]. The BART assesses the willingness to consciously risk long term loss in order to get higher rewards on the short term [67]. A Dutch adaption of the task by C. Nederkoorn was used. This version of the BART is a computer task consisting of a series of 29 trials. In every trial a small red balloon is shown on the computer screen. The participant is instructed to inflate the balloon by pressing a pump button. He or she is warned that the balloon will explode at some point, but that this can happen any time from after the first pump until after the balloon has expanded to fill the whole screen. Every pump produces a gain of 5 points in a temporary bank. As long as the pumping continues, the points go up. However, the balloons all explode at some point, according to a predetermined sequence with the weakest balloon exploding on the first pump and the strongest balloon exploding after 128 pumps. If that happens before the gained points are transferred to the permanent bank, all points for that balloon are lost. Transfer to the permanent bank can be done at any chosen moment by pressing the collect button and results in the next trial with a new uninflated balloon. The total amount of points earned is shown on the computer screen [65]. In deciding whether to make each pump, the participant must balance the potential gain of accruing more money against the potential risk of losing all money accrued for that balloon [71]. Individuals who are more sensitive to rewards will demonstrate a higher number of pumps on each balloon prior to money collection [65]. For analysis of the results the adjusted value is used, defined as the average number of pumps excluding balloons that exploded [65,72,73].

##### Self-report measure of eating specific self-regulation ability

In addition to the computer tasks, self-reported indicators of eating-specific self-regulation are assessed. Hereto, a version specifically adapted to children [74] of the Dutch Eating Behavior Questionnaire (DEBQ), originally developed by Van Strien and colleagues [75], is used. This 33-item questionnaire assesses three eating styles that reflect dysfunctional regulation of food intake: 1) external eating (i.e. the tendency to eat in response to external cues), 2) emotional eating (i.e. the tendency to overeat in response to emotions) and 3) restrained eating (i.e. the tendency to attempt to refrain from eating) [75].

#### Moderators of the relation between self-regulation ability and weight loss and weight loss maintenance

As outlined earlier, factors related to competence, motivation, relatedness and outcome expectations will be examined as moderating factors. Hereto, all moderating factors are assessed at baseline and at follow-up examinations. Except for a few moderators that are only measured at T0.

##### Competence

Self-efficacy is measured with the Dutch adaptation of the 10-item General self-efficacy (GSE) scale, which assesses one’s sense of personal competence to cope across a variety of demanding or novel situations [76].

Self-worth is measured with Dutch adaptations [77,78] of the Self-Perception Profile for Children (SPPC) and Adolescents (SPPA) developed by Harter [79,80], which are questionnaires with 36 items (SPPC) or 35 items (SPPA) in six (SPPC) or seven (SPPA) domains: 1) global self-worth, 2) scholastic competence, 3) social acceptance, 4) athletic competence, 5) physical appearance, 6) behavioral conduct, and 7) close friendship (only SSPA).

##### Motivation

Motivation to enter a weight loss program is measured with a 18-item version of the Treatment Self-Regulation Questionnaire (TSRQ) that assesses reasons to enter a weight-loss program [81]. The questionnaire was translated into Dutch by two of the authors of this article (JH and EdV) with help from a native English speaker, in which process it was slightly adapted for use with children and adolescents. It assesses to what extent children and adolescents enter a weight-loss program for autonomous (e.g. “I really want to make some changes in my life”) and controlled reasons (e.g. “People will like me better when I’m thin”) on a 7-point Likert scale ranging from 1 (not at all for this reason) to 7 (totally for this reason).

##### Relatedness

Interaction between child and parent is measured with the Dutch version of the Parent–child Interaction Questionnaire – Revised (PACHIQ-R) [82] that assesses how the parent evaluates the relationship with the child (PACHIQ-R Parent version, 21 items) and how the child assesses the relationship with the parent (PACHIQ-R Child versions for mother and father, 25 items). The questionnaire concerns both behavioral interaction and attitudes. It has two subscales: conflict resolution (quality of preventing and solving conflicts) and acceptance (warmth, comfort, protection).

Social competences and social problems as assessed by the parent are measured with the Child Behavior Checklist (CBCL/6-18) [83] that rates children’s functioning. We use the Dutch translation [84] of the questionnaire that assesses competences (16 items) and behavioral and emotional problems (120 items) in the past six months. In the present study, only two scales are included: 1) The “Social scale” (this is one of the three competence scales) that comprises 6 questions, which concern group activities and social relationships (e.g.: “Please list any organizations, clubs, teams, or groups your child belongs to”; “About how many close friends does your child have? (Do not include brothers & sisters)”; “Compared to others of his/her age, how well does your child play and work alone?”) [83]. 2) The “Social problems scale” (this is one of the five emotional and behavioral problem scales) that consists of 11 items, which concern topics like loneliness, jealousy, getting teased and clumsiness (e.g.: “Clings to adults or too dependent”; “Complains of loneliness”; “Easily jealous”; “Feels others are out to get him/her”) [83].

Parental feeding style is measured with a Dutch translation [85] of the 27-item Parental Feeding Style Questionnaire (PFSQ) which asks parents to report the frequency with which they use a number of feeding strategies that are grouped into 1) emotional feeding (i.e. “I give my child something to eat to make him/her feel better when s/he is feeling upset), 2) instrumental feeding (i.e. “I reward my child with something to eat when s/he is well behaved”), 3) prompting/encouragement to eat (i.e. “I praise my child if s/he eats what I give him/her”) and 4) overt control over eating (i.e. “I decide how many snacks my child should have”) [86].

Affect of the parents is measured with the Dutch translation of the 20-item Positive and Negative Affect Schedule (PANAS) [87,88], which asks parents to indicate what they currently feel on two scales: 1) positive affect (the extent to which a person feels enthusiastic, active and alert) and 2) negative affect (subjective distress and unpleasurable engagement that subsumes a variety of aversive mood states, including anger, contempt, disgust, guilt, fear and nervousness).

Peer body size is measured by asking children and adolescents to select from a range of line drawings (separate for boys and girls) the silhouette that most closely resembles how their five best same sex friends look. The used drawings are taken from Colleen S.W. Rand [89], who composed the drawings based on drawings by M.E. Collins [90] and T.I.A. Sorensen et al. [91].

##### Outcome expectations

The same silhouette drawings are also used to assess outcome expectations. Children and adolescents are asked to select 1) the silhouette that most closely resembles their current body size and 2) the silhouette that most closely resemble the body size they expect to have after treatment. Outcome expectations are calculated by measuring the difference between these current and expected own body sizes.

#### Covariates

Several covariates will be assessed. It is likely that the intensity and duration of treatment are related to both the ability to self-regulate and ΔSDS-BMI. We will investigate the intensity of the treatment, expressed by the duration of the residential period (2 versus 6 months) as it might explain part of the variation between individual children and adolescents in weight loss and in the ability to self-regulate at the end of treatment (T12). We will also investigate the health care the children and adolescents receive in the period between the end of the treatment at Heideheuvel and the follow up one year later as the prolongation of treatment might influence weight loss maintenance after the first year of treatment. Furthermore we will assess the gender and age-specific ΔSDS-BMI between baseline (T0) and end of treatment (T12) because the amount of weight lost during treatment might be a predictor of weight loss maintenance after treatment. Finally the influence of SDS-BMI at the start of treatment, gender, age and educational level of the children and adolescents on the long-term weight outcome will be assessed.

### Statistical analyses

First we will provide information on the baseline characteristics of the patient group. Secondly, SDS-BMI levels and changes will be described at the three time points, i.e. at baseline (T0), after one-year (T12) and at two-year follow-up (T24). This allows for the description of total weight loss (computed as ΔSDS-BMI between T0 and T24) and weight loss maintenance (computed as ΔSDS-BMI between T12 and T24). Next, determinants (general and eating-specific self-regulation) and moderators (factors related to competence, motivation, relatedness and outcome expectations) will be described and compared at baseline (T0) and one year later (T12). This enables detailed scrutiny of the variability of the determinants and moderators in this specific target group of severely obese children and adolescents. Furthermore, it will provide insight into the change in determinants and moderators after following a treatment of one year.

Third, the relation between self-regulation and initial weight loss at one-year follow-up and potential moderators of this relation will be tested using hierarchical multiple regression analyses. Linear regression analyses (with ΔSDS-BMI between T0 and T12 as dependent variable) and logistic regression analyses (with a dichotomous variable high versus low weight loss as dependent variable) will be conducted. To discriminate participants with a high and low weight loss a median split in ΔSDS-BMI between T0 and T12 will be conducted. In the first step, other factors will be added (e.g., age, gender, SDS-BMI at T0, treatment intensity). In the second step, the self-regulation indicators (at T0) will be entered simultaneously as independent variables. In the third step, interaction terms between the moderators (at T0) and (the most important) self-regulation indicators will be computed and added to the model. Significant interaction terms will subsequently be decomposed to gain insight into the relation between self-regulation and initial weight loss at high and low levels of the moderator.

Finally, the relation between self-regulation and weight loss maintenance at two year follow-up and potential moderators of this relation will again be examined using hierarchical multiple regression analyses. Linear regression analyses (with ΔSDS-BMI between T12 and T24 as dependent variable) and logistic regression analyses (with a dichotomous variable high versus low maintenance of weight loss as dependent variable) will be conducted. To calculate high versus low maintenance of weight loss a median split in ΔSDS-BMI between T12 and T24 is conducted. The same steps as in the one-year analyses will be conducted, except that for the determinants and most moderators the data collected at T12 will be entered. A few moderators that are only measured at T0, will be entered as assessed at T0.

We will explore the possibility of multiple imputations to deal with missing data. Missing data will be imputed multiple times, using the ‘Predictive Mean Matching’ (PMM) procedure that runs under the SPSS 20.0 program. All analyses will be performed on the complete case data and on the imputed data. In case of missing item scores, we will explore the possibility of a single imputation technique.

## Discussion

We have identified psychosocial factors predicting weight loss success from studies done in (obese) adults. Whether or not these are also determinants in younger patients suffering from severe obesity, is not known. The present study aims to fill this gap.

This study will provide knowledge about the relation between self-regulation and long-term weight loss after intensive lifestyle interventions in severely obese children and adolescents, a growing but often overlooked patient group. We aim to investigate to what extent (changes in) the general ability to self-regulate predicts weight loss and weight loss maintenance. This study will also contribute to the knowledge on how this association is modified by other psychosocial factors. The results can provide important information about the potential for treatment success according to psychosocial characteristics. This may help professionals to tailor interventions to children. The information can also be used to determine which patients might need more guidance than others in the relapse prevention phase after an intensive intervention.

The study population is severely obese and has not been able to profit sufficiently from previous treatments, which may suggest that these patients are relatively therapy resistant. It remains to be investigated whether this is really the case. If so, the present study might result in a lack of variance in weight loss success and maintenance which would limit the analyses. That the study population belongs to a very specific subgroup of patients also implies that the study results cannot easily be generalized to other treatment populations or other treatment forms. Interpretation of the study results should also be done with caution because the data collection on what happens to the patients in the follow-up year is limited to measurements at the end of follow up. The effect of for example major live events in this period is not included in the analyses.

In addition to these limitations, also some important strengths need to be acknowledged. Studies with a long term follow up are scarce. This study, with a follow up of 2 years, is relatively long. Short-term weight loss is much easier to achieve than longer term weight loss and weight loss maintenance. Our primary endpoint (2 years post-baseline) strikes the balance between following the patients and their parents long enough to assess long-term outcomes with what is feasible within a 4 year study period. The two year time span gives us the opportunity to assess the effects of the therapy after its termination. Moreover it also enables the investigation of maintenance of the attainted ability to self-regulate. Nevertheless we are not able to follow the participants’ health and wellbeing for life and the long-term treatment impact remains speculative. They suffer from a chronic condition that can result in various medical, psychological and social difficulties during the course of their lives. In order to make measurements after a longer follow up period possible, all participants in the study are asked during the last measuring moment whether they are willing to participate in future research.

A final issue that needs to be discussed is the use of SDS-BMI and behavioral computer tasks as proxies for change in dietary intake. Generally, instruments to capture dietary intake may not be sufficiently valid. A healthier dietary pattern is assumed to be reflected in a change in SDS-BMI. In a similar vein, improvements in self-regulation abilities (as assessed with the computer tasks) are assumed to echo in improvements in dietary intake. Computer tasks are used because they provide a relatively objective measurement of self-regulation abilities. Both used computer tasks are non-food related. They measure general self-regulation, a supposedly general ability that affects eating behavior. If the results of the computer tasks do not show a relation between self-regulating ability and weight loss maintenance, this might be attributed to the use of these general measuring instruments and does not necessary mean there is no change in self-regulation of eating behavior. If this study does show an association between self-regulation ability and weight loss maintenance, future research could extend on that by demonstrating the causal pathway explaining the association.

## Conclusion

To conclude, the present study will provide knowledge about the role of self-regulation abilities in weight loss success and maintenance in severely obese children and adolescents over a two-year period.

## Competing interests

OHBS is affiliated with the treatment program as pediatrician. Other than that the authors declare no competing interests.

## Authors’ contributions

All authors made substantial contributions to the conception and design of the study. JH, EV and JCS drafted the manuscript. All authors were involved in revising the manuscript critically for important intellectual content. All authors read and approved the final manuscript.

## Pre-publication history

The pre-publication history for this paper can be accessed here:

http://www.biomedcentral.com/1471-2431/13/41/prepub
